# Provings and Clinical Observations with High Potencies

**Published:** 1893-05

**Authors:** Malcolm Macfarlan

**Affiliations:** Philadelphia


### PROVINGS AND CLINICAL OBSERVATIONS WITH HIGH POTENCIES.

MALCOLM MACFARLAN, M. D., PHILADELPHIA.

### KALI-HYD.$^{CM}$.

Confused, senseless, unconnected dreams the whole night, causing unrefreshing sleep; cannot remember his dreams.

### PALLADIUM$^{20M}$.

Wakeful until two o'clock in the morning; a very prominent symptom.

### PHYTOLACCA$^{47M}$.

Sleepy, in a slight degree.

### PLANTAGO-MAJ.$^{5C}$.

After supper broke out in a profuse sweat, and then felt so sleepy couldn't keep awake; awoke in a fright, a symptom observed in several provers.

### POLYGONUM-HYDROP.$^{42M}$.

Sleepless; not a prominent symptom.

### RHUS-TOX.$^{105M}$.

Cannot sleep at night for the itching of the feet; *restlessness very great*, because of general irritation of the skin; involuntary scratching.

### SAMBUCUS-NIG.$^{45M}$.

Night-mare; vivid, frightening dreams; dreams of drowning.

### FEVER.

### ACONITE$^{50M}$.

Has frequently arrested for a time the night-sweats of consumptives; usually causes profuse sweat; profuse sweat during sleep, and for some time after waking; several provers had to get up in the night to change their clothing in consequence; cured or modified great sweating in a variety of diseased conditions; generally causes rapid decrease in temperature in acute febrile conditions. In the judgment of the writer it is the most useful remedy for this purpose.

Sweating is the first symptom or most prominent after giving potentized Aconite.

### ARSENIC$^{6M}$.

Chilly and feverish, mostly toward sundown; *night-sweats;* cannot sleep very well because of mental anxiety, which is apparently without cause; fever and sweat every afternoon; sweat again late in the night; it is general or over the whole body, but not profuse.

### AGARIC-MUSC.$^{47M}$.

Continued thirst, unquenched by water or liquids; very marked symptom; usually drinks nothing between meals, now takes a gread deal of water; kidneys very active, but apparently has no fever.

### ASCLEPIAS-TUB.$^{45M}$.

Free perspirations without fever; continual slight moisture of skin.

### Bursa-pastoris$^{9M}$.

Violent fever, very thirsty; fever lasts but a short time, followed by free perspiration.

### Cimicifuga-rac.$^{95M}$.

Slight fever every afternoon between twelve and four.

### Cornus-flor.$^{45M}$.

Chilly sensations come on frequently, although the prover is generally warm at the time or does not wish to be covered; so-called nervous chills.

### Eupat-perf.$^{CM}$.

Attacks of chills in the morning; could not get warm, so chilly; slight fever, followed by very free perspirations.

### Guajacum$^{5C}$.

Violent fever; face became spotted during the fever; eyes, nose, and cheeks appeared puffy; tight, dry cough.

### Hydrophobin$^{1M}$.

Much fever; no preceding chill; pulse small and rapid; great thirst; great desire to drink quantities of cold water.

### Hyosc-nig.$^{CM}$.

Ague-like chill every other day at eleven o'clock; fever and sweat, with continual *stupefying headache;* cannot bear the least noise and avoids the light.

### Iodine$^{17M}$.

Considerable fever; hot, dry, burning skin all day long; creepy sensations; no sweat.

### Oxalis$^{1M}$.

Sudden flushes of heat, alternating with nervous, chilly sensations.

### PHYTOLACCA-DEC.$^{47M}$.

Chilly every morning, in a mild degree.

### PLANTAGO-MAJ.$^{5C}$.

Free perspiration in the early part of the evening, preceded by slight fevers.

### PLANTAGO-MIN.$^{5C}$.

Fever of a mild type comes on suddenly, followed by sweat; nervous, frightening dreams; all this in the fore-part of the night.

### NAT-MUR.$^{CM}$.

Causes general coldness; disposition to put on more clothing; cured very many cases of chills and fever, especially coming from the prevailing practice, and after the use of Quinine. Chilliness without much if any sweat.

### SYMPHYTUM-OFF.$^{15C}$.

Alternately cold and feverish all day; after a few days continued coldness and a desire to have on more clothing.

### CHELIDONUM-MAJ.$^{5C}$.

Chilliness with nausea, mostly in the morning.

### TROMBIDIUM$^{5C}$.

Occasionally flushes of heat would come on suddenly and disappear in a similar manner.

### WOORARI$^{90M}$.

Extremities appear blue during fever; after the fever passes away patient becomes very red, mostly in the face. This was a marked and singular symptom. The circulation seemed much disturbed.

### SAMBUCUS-NIG.$^{45M}$.

Much fever; continual thirst; perspiration not great.

### THERIDION-CURASS.$^{5C}$.

Chilly; patients described their bones as being sore.

TEREBINTH$^{17M}$.

Craves fluids; continual thirst and some slight fever, without chill or sweat; abdomen is fuller than usual from excess of gas.

SECALE-CORN.$^{95M}$.

Very thirsty; wants to drink continually; severe chill, lasting from four A. M. till ten A. M., felt mostly in the small of the back, extending along the spine; when having the chill, shooting pains occurred in his legs and arms; very thirsty during the chill, not much during or after the fever; slight sweat; mouth dry; violent nausea; vomited a great quantity of bile; superficial congestion; veins of extremities very full.

CAUSTICUM$^{30M}$.

Shivered constantly night and day; could not attend to work because of it; a very prominent symptom.

NATR-MUR.$^{CM}$.

Has proven highly curative in chilly sensations and intermittent fever occurring under a variety of diseased conditions.

### MIND.

APOCYNUM-CANN.$^{6M}$.

Feels as if she could do nothing but cry; does cry; very low-spirited; does not want to speak to her friends. This is not because of her sex particularly, or because of an easily affected disposition; I believe it due to the remedy. Sadness.

APIS-MELL.$^{5C}$.

Great tearfulness. Females taking this remedy easily moved to tears.

APIS-MELL.$^{CM}$.

Believed to have cured my child, which had serious brain congestion and inflammation, attended with occasional screaming attacks; partial suppression of urine; child, before the exhibition of this remedy, had been in a partial stupor for three or four days, and had been sick two weeks with very alarming symptoms; the relief was sudden and remarkable.

### ARSENIC-ALB.$^{6M}$.

Fearful depression of spirits; wants to go to a hospital, because of expense; thinks of nothing but impending danger. Another wants to run away, leave; dreads that something horrible will happen; restlessness in all the cases; sadness; apprehension. Fright, fear, and sadness more prominent symptoms than in any other remedy known to the writer.

### ARUM-TRIF.$^{16M}$.

Nervous and anxious in a slight degree.

### BELL.$^{101M}$.

Given to a man nearly dead with consumption; set him crazy; he got partly out of the window, intending to kill himself, and had to be tied down in bed; exceedingly cross, ill-tempered; disposed to be violent and pugnacious; uneasy, restless. In many other cases mild mania and hallucinations.

### CALEND-OFF.$^{45M}$.

Feels as if he would fall from a height, when dropping to sleep.

### CHINA-OFF.$^{87M}$.

*Exceedingly nervous;* wandering neuralgic pains throughout the body; slight, unexpected noises or appearances cause starting.

### CORNUS-FLOR.$^{45M}$.

Cured an irritable temper and vexed state of long standing, guided by the symptom of extreme melancholy produced by this remedy.

### EUPAT-PERF.$^{CM}$.

Feels as if he were going out of his mind. This symptom seems to overwhelm him at night.

### Psorinum$^{42M}$.

Nervous; easily startled; a condition very marked in children to whom the medicine was given.

### Scutellaria-laterif.$^{40M}$.

Fearful nervousness; restlessness; it has a marked curative effect in relieving the sleeplessness of excitable people of an amiable and sensitive nature, women and children particularly; in them it has promptly induced sound, refreshing sleep. Its remarkable soothing effect was discovered accidentally.

### Saccharum-lac.$^{CM}$.

Caused fullness and determination of blood to head; dizziness; cured the latter symptom, which had been of very long standing.

### Woorari$^{90M}$.

*Instant giddiness.* This condition appeared frequently and had no bad attending or following symptoms; felt for a moment as if he was bereft of his senses.

### HEAD.

### Æsculus-hipp.$^{56M}$.

Frontal headache of a mild type.

### Agaric-musc.$^{47M}$.

*Beating on top of head* and toward the forehead.

### Aletris-farin.$^{45M}$.

Slight, dizzy headache.

### Apium-virus.$^{69M}$.

Dull aching in forehead.

### Apis-mell.$^{85M}$.

Scalp sensitive.

### Apocynum-cann.$^{6M}$.

Frontal headache, fullness in forehead; ideas confused; inability to think well.

### Arum-trif.$^{16M}$.

Light-headed, giddy; not headache.

### Ailanthus$^{45M}$.

Intolerable itching, with sore spots about the head and face.

### Arsenic-alb.$^{6M}$.

Headache on top of head.

### Baptis-tinct.$^{6M}$.

Nape of neck feels so tired she can hardly keep her head easy in any position.

### Bell.$^{101M}$.

Rouses up and screams; starts and screams, as if frightened; does not wish to sit up in bed.

### Benzoic-acid.$^{11M}$.

Guided by symptom of turbid, offensive urine; cured severe pain in occipital region, which had confined him to bed for several weeks; produced soreness in scalp and nape of neck in several provers.

### Borax$^{3M}$.

Pain across forehead, lasting several days continuously and with great giddiness, pain extended to top of head, for a short time; soreness along both sides of the neck.

### Bromine$^{CM}$.

Headache all week; hammering in temples and top of head.

### Bursa-pastoris$^{9M}$.

Intense frontal headache.

### CAUST.$^{30M}$.

Feels as if there was great heat in the forehead.

### CALENDULA-OFF.$^{45M}$.

Splitting headache in frontal region.

### CAPSICUM-ANN.$^{1M}$.

Severe headache all over the head, coming and going frequently; headache is relieved by being in the dark or closing the eyes.

### CICHOR-INTYB.$^{5C}$.

Neuralgia down the sides of the neck; more on the right side; pain suddenly attacks him in muscles of the neck, like a stitching sensation.

### CAUSTICUM$^{30M}$.

Severe pain at each parietal protuberance, internal as well as external.

### CUPRUM-ACET.$^{45M}$.

Slight headache in the temples, worse at *junction of* occipital with both parietal bones; aching pain, with soreness of scalp to the touch.

### DOLICHOS-PRUR.$^{2C}$.

Bones of the head feel as if they would separate; headache is so severe. This is the language of the prover.

### ETHER-SULPHURIC$^{CM}$.

Remarkable effect in curing chronic neuralgia of the head, which kept her in bed for weeks; swelling of scalp after attacks of pain; complete prostration. These attacks, which came on several times a year, were cured after giving this remedy.

### GINSENG.$^{46M}$.

Sharp pain at the top of head; relieved by closing the eyes or avoiding light.

### HAMAMELIS-VIRG.$^{10M}$.

Knocking, hammering over the left eye; felt as if he was going out of his mind; flushed face; sensation of fullness in head.

### FLUORIC-ACID$^{45M}$.

Pain through the temples, sharp or acute.

### FELIX-MAS.$^{1M}$.

Giddiness.

### IODINE$^{17M}$.

Pain in occiput and temples.

### IRIS-VERS.$^{CM}$.

Cured chronic neuralgic headache with asthenopia, and severe dyspeptic symptoms; distress at the epigastrium; flatulency.

### KALI-CARB.$^{24M}$.

Stiffness in nape of the neck.

### KALMIA-LATIF.$^{12C}$.

Very violent, blinding neuralgic headache; severe pain over the left eye; giddiness; nausea.

### KREOSOTE$^{CM}$.

Cured very many chronic or sick headaches, with piercing pain through the temples; sensitive and slightly raised scalp. These cures were made in both sexes, and where many remedies had been used without avail. Pimples about the face of most of the patients.

### LEDUM-PAL.$^{45M}$.

Sensation as if something were gnawing within her temples, back of her ears and occiput.

### LILIUM-TIG.$^{45M}$.

Mentally confused and heavy; glands on left side of neck swollen and painful.

### LACHESIS$^{CM}$.

Violent pain all over his head, back and front; so giddy he could not stand; had to be carried from school; could not see the letters in his book; fell against the wall; this occurred on the fourth day; taking medicine every hour; he never had these symptoms before; one prover. The symptom giddiness had often before been observed in other provers, accompanied by disposition to swallow or slight choking sensations.

### LAMIUM-ALB.$^{1M}$.

Severe congestive headaches in the morning; giddy, worse on left side; general muscular soreness.

### MERC-VIV.$^{101M}$.

Great pain in the *forehead;* back and upper part of the head affected slightly.

### MERC-IOD.$^{2C}$.

Pressive pain in forehead, aggravated toward night.

### MEZEREUM$^{103M}$.

Highly curative in severe nervous frontal headaches. One of the most beneficial remedies in ciliary pains, with glaucomatous symptoms; headache, with great intolerance of light.

### MISSISSQUOI-WATER$^{45M}$.

Neuralgia in head; sensation as if suffering from cold in head, nose, and air passages.

### MURIATIC-ACID$^{5C}$.

Sensation of heat at the top of his head; slight neuralgia on the left side.

### NITRIC-ACID$^{5C}$.

Giddy; head feels hot; continual slight aching all over the head.

### Nux-vom.$^{94M}$.

Aching at the top, which extends to the back of the head and neck; given to those ailing, produced increased ability to think with less nervousness.

### Palladium$^{20M}$.

Headache across the top of his head, from one ear to the other; pain most severe at the right parietal protuberance; giddy when lying down.

